# Not only Iranian rise in science marred by fraud: Misconduct is a global problem

**DOI:** 10.17179/excli2017-263

**Published:** 2017-08-15

**Authors:** Bagher Larijani, Kamal Niaz, Ata Pourabbasi, Fazlullah Khan, Jonathan Spoor, Mohammad Abdollahi

**Affiliations:** 1 Endocrinology and Metabolism Research Center, Endocrinology and Metabolism Clinical Sciences Institute, Tehran University of Medical Sciences, Tehran, Iran; 2 International Campus, Tehran University of Medical Sciences (TUMS-IC), Tehran, Iran; 3 Toxicology and Diseases Group, Pharmaceutical Sciences Research Center, Tehran University of Medical Sciences, Tehran, Iran; 4 Research Center for Immunodeficiencies, Pediatric Center of Excellence, Children's Medical Center, Tehran University of Medical Sciences, Tehran, Iran; 5 Erasmus University Medical Centre, Erasmus University Rotterdam, Rotterdam,the Netherlands

## ⁯

Dear Editor,

We regard the fact that Science magazine is a non-political and impartial scientific journal, which publishes prestigious news and papers, contributing to the development and expansion of scientific knowledge, as universally recognised. However, in the volume of September 2016, we perceived a news report titled: “A shady market in scientific papers mars Iran's rise in science” written by Stone (Stone, 2016[8]). In that report, the author raised the issue of the production of scientific papers and theses in specific shops around the University of Tehran, Iran. He also purported that some of these 'counterfeit' theses find their way to reputable international journals for publication. Real scientists, he asserted, are not involved in these shady dealings. He mentioned that the sole purpose of that business is earning money. According to Stone, one can easily get a copy of a thesis related to almost any discipline just by paying money. Although university faculty members supposedly are not part of such market-based publications, there are certain non-faculty experts who have established facilities around the university where scientific papers are written for the sole purpose of financial gain. Reading this report, some considerations came to mind which we would like to share with the readers. From our point of view, it would be appropriate to bring some nuances to the image that was created by the writer of the concerning article. That is why we decided to write this letter to bring to the attention of the readers some important points.

First of all, we fully agree with the view of the news author that shady market practices in the field of science have a mutilating effect and is not acceptable at all. However, our criticism is directed at the methodology through which he identifies this issue. According to us, this problem is not limited to Iran or China and it is a worldwide phenomenon. Students wish to use online softwares in their studies and research, write clean theses, and if possible, publish their papers. Sadly, certain 'predatory' companies have found that this desire creates a demand that they can exploit to earn money. We do not disagree about the fact that a shady market of science might exist in Iran, but we would like to criticize the report in terms of its bias.

Stone's report lacks on the following points:

- Insufficient people's quotations are not appreciated.
- The bibliographic search strategy is not clear.
- Systematic evaluation of the sample size is necessary.
- Mentioning of a particular image, which had been taken in the street around the University of Tehran shows ambiguous assumption.
- The scientific communities in two of the world's oldest civilizations, namely China and Iran, are swiftly denounced in the light of highly selective reports of unethical scientific conduct.
- In good scientific practice, referral to only one report of market-based publications is insufficient to address this issue.

Regardless of the image that Stone (2016[8]) creates in his article, scientific deceit presents itself globally and not just in China or Iran. For instance, drug companies advertise their products with claims about safety. Nevertheless, patients might lose their lives due to production errors or toxic side effects. Certification of biological products does not prevent the appearance of duplicates on the market, which deceive the consumer. Apart from these examples of dupery in the field of medicine, there is a global scientific swindle going on that transgresses the borders of any scientific discipline. There are several fraudulent websites on the worldwide web that cheat scholars and students into paying for services that ultimately are never delivered (Jalalian, 2015[5]). These websites are being brought on the internet from a variety of locations worldwide. As mentioned before, it is the ambition of students, and researchers to publish articles to upgrade their academic records, to obtain grants/scholarships, and sometimes provide the mandatory requirement of the universities to graduate. Sometimes, scientists and students have to work under the pressure of some academic rules to publish more (Abdollahi et al., 2014[1]), a matter that we cannot agree with. This may lead to the temptation to adopt questionable strategies. By some websites that put themselves forth as publishers, they are led to believe that they obtain publications in reputable journals, while in reality their papers are published in fake and fraudulent journals. Fabrication, duplication, falsification, plagiarism, fake authorships and data manipulation are the kinds of misconduct that are employed around the globe (Asghari et al., 2017[2]). Nevertheless, an ethical and well-informed academic environment is of unequalled importance in the development and distribution of scientific ideas. In the new era of science, the quality of research is measured against the impact factor of the journal in which it is published. This has made academic publishing from a deliberate but slow process to an open-access model. Sadly, cyber offenders are responding to this global development in a very smooth way (Saeidnia and Abdollahi, 2015[6]). The past decade has seen the formation of a myriad of new publishers that have published a huge number of articles perhaps without proper peer-review or regard for ethics. They are no more than a commercial party responding to the needs of the academic society, which it has unravelled very cleverly. All that is needed for this lucrative scientific swindle is to elaborate a computer software, a well-developed framework, web development, email marketing, and prey collections. By their conduct these 'predatory' publishers smear the reputation of many companies that help researchers in an ethical way by performing statistical analysis, proofreading or by finding a publisher (Gasparyan et al., 2017[4]; Jalalian, 2015[5]). 

From various points of view, most of Iranian scientists conduct impressive and clean studies as they publish their works mainly in legitimate and peer-reviewed sources. By scrutinizing the SCImago journal and country ranking database (www.scimagojr.com[7]) however, we can find that Iran has jumped from 53^rd^ to the 16^th^ position in the period from 1996 to 2016 in all subjects areas, categories and regions. The same website puts Iran on the first position in the Middle East. In 2016 worldwide ranking, Iran has been in the 8^th^ position in Pharmacology, Toxicology, and Pharmaceutics, 12^th ^position in Dentistry, 12^th^ position in Chemistry, 15^th ^position in aerospace engineering, and 18^th^ position in Medicine, etc. These achievements can only be obtained by delivering inspiring and influential scientific work, although Iran has been under tough international sanctions during the past decades, affecting almost every scientific discipline. Therefore, according to us, it would be out of turn to underestimate the invaluable advancements made by Iranian scholars and researchers in science and technology. There has been a dramatic increase in the university population of Iran from 100,000 in 1979 to about 2 million in 2006. Furthermore, women have acquired a vital role in the contemporary development of the sciences in Iran. Nowadays about 70 % of its science and engineering students are women.

Regarding Stone's report, the educational system of Iran (population exceeding 80 million) cannot be judged by walking the sidewalks of its capital Tehran. He presumes that roughly 10 % of all masters and PhD theses in Iran may have been obtained from dealers. However, he fails to appreciate the fact that unethical and fraudulent practices are not limited to the borders of Iran. Noncompliance with rules may occur in every country and these issues are not limited to Iran or China (Asghari et al., 2017[2]). Worthy to mention, the difficulties Iranian scholars have faced with financial and logistical obstacles due to sanctions in the past 3 decades and its negative impact on the growth of science should not be missed (Astaneh, 2016[3]). Hopefully, Iran has developed strong ethical committees countrywide in all academics to deal exactly with the issues that Stone mentions. The Iranian government is in the process of launching the new rules on complying with ethics codes in academics that prohibits any misconduct and assigns serious penalties for any violation of the rule. 

We believe that Stone's (2016[8]) report, is somehow biased and gives a distorted image of the way science is conducted in Iran. This might have caused some undue confusion to readers. Stone has failed to appreciate the fact that findings on his strolls through certain parts of Tehran cannot overnight be extrapolated to a whole country. Furthermore, he forgot to mention the limitations of his report that includes but one reference and a few quotations. Extrapolating the existence of thesis and research forgery to an all-encompassing trend in the countries' rise in science, as Stone does in the title of his article, therefore is unjustified.

## Author contributions

All authors have directly participated in planning or drafting of the manuscript and read and approved the final version.

## Conflict of interest

The authors declare no conflict of interest.

## References

[1] Abdollahi M, Gasparyan AY, Saeidnia S (2014). The urge to publish more and its consequences. Daru.

[2] Asghari MH, Moloudizargari M, Abdollahi M (2017). Misconduct in research and publication: a dilemma that is taking place. Iran Biomed J.

[3] Astaneh B (2016). Iran’s scientific community shouldn’t be put in the shade. thebmjopinion.

[4] Gasparyan AY, Nurmashev B, Udovik EE, Koroleva AM, Kitas GD (2017). Predatory publishing is a threat to non-mainstream science. J Korean Med Sci.

[5] Jalalian M (2015). The story of fake impact factor companies and how we detected them. Electronic Physician.

[6] Saeidnia S, Abdollahi M (2015). Peer review processes and related issues in scholarly journals. Daru.

[7] SCImago journal and country ranking database www.scimagojr.com.

[8] Stone R A shady market in scientific papers mars Iran’s rise in science. Science.

